# Synthesis of thiol derivatives of azobenzocrown ethers. The preliminary studies on recognition of alkali metal ions by gold nanoparticles functionalized with azobenzocrown and lipoic acid

**DOI:** 10.1007/s10847-015-0567-y

**Published:** 2015-09-25

**Authors:** Elżbieta Luboch, Mirosław Szarmach, Anna Buczkowska, Ewa Wagner-Wysiecka, Magdalena Kania, Witold Danikiewicz

**Affiliations:** Department of Chemistry and Technology of Functional Materials, Faculty of Chemistry, Gdańsk University of Technology, Narutowicza 11/12, 80-233, Gdańsk, Poland; Institute of Organic Chemistry, Polish Academy of Sciences, Kasprzaka 44/52, 01-224, Warsaw, Poland

**Keywords:** Azobenzocrown ethers, Thiol compounds, Gold nanoparticles, Potassium-selective nanosensor

## Abstract

The article presents the synthesis of novel 13- and 16-membered azobenzocrown derivatives with peripheral thiol moieties and preliminary studies assessing their possible application in plasmonic sensors based on gold nanoparticles. The effect of the length of the chain connecting the macrocycle with the thiol group and the effect of the presence of the additional functional compound, i.e. lipoic acid, on the sensor response was analyzed. Colloidal gold nanoparticles modified with a 16-membered crown with a thiol group on oxyethylene (compound **12**) or oxybutylene (compound **13**) linker was found to have good properties, allowing for detection of potassium ions in aqueous solutions at concentrations 8-20 mM for bifunctionalized nanogold and 4-26 mM for less stable, colloidal gold modified only with thiol derivatives of azobenzocrowns. The response towards potassium cations of bifunctionalized nanogold modified with compound **13** was more stable in time than for the system incorporating compound **12**. Compound **13,** obtained with the highest yield among all presented thiol derivatives of azobenzocrowns, was selected for further, more detailed, studies.

## Introduction

The rapid advances in the field of nanotechnology are associated, among other factors, with the possibility to design new materials, subcomponents and systems having unique properties. The interest in this field is high due to e.g. the possibility to use the nanosystems in miniature optical devices, sensors or medical applications.

Not only nanotechnology but also supramolecular chemistry is presently one of the most active fields of science [1]. One of the areas of interest in supramolecular chemistry is artificial receptor molecules. For example, crown ethers are well-known metal cation complexing macrocyclic agents [2]. Interesting group of macrocyclic compounds are azophanes [3, 4]. Azophanes are crown ethers with azo groups constituting a part of the macrocycle, formed by incorporating the azo group, most commonly an azobenzene fragment substituted in various positions, into the macrocycle. The azobenzene moiety built into the macrocycle, along with its possible participation in complexation processes, may serve additional purposes, such as the control of ion complexation by *trans*–*cis* isomerization [5, 6].

Azobenzocrowns, derivatives of 2,2′-substituted azobenzene, containing different substituents, turned out to be universal metal cation hosts from the analytical point of view. Depending on the nature of the substituents in the benzene rings, azobenzocrowns can act as chromoionophores, enabling studies of their interactions with metal cations using UV–Vis spectroscopy [7–9], or as an effective ionophores in membrane ion-selective electrodes (ISEs) [10, 11].

Currently, much attention is focused on plasmonic nanosensors [12–17]. In this respect, systems based on gold nanoparticles (GNPs) appear to be particularly interesting. They take advantage of the fact that red/purple colloidal solutions containing GNPs become blue upon aggregation, which can be observed as the shift of the surface plasmon resonance (SPR) band in the absorption spectrum. The optical properties of these systems depend on the shape and size of nanoparticles [18–22].

There are many methods of preparation of GNPs [23]. The most popular and the longest-used method is the reduction of HAuCl_4_ with citrates in water. Citrate, besides acting as a reducing agent, stabilizes the formation of the colloidal system [24]. The method for determination of the reagent ratios required to obtain nanoparticles of desired size is based on the relationship between the nanoparticle diameter and the molar ratio of citrate within the mixture [25]. The synthesis of colloidal spherical GNPs based on the reduction of tetrachloroauric acid with citrates in aqueous environment was subjected to a number of modifications [26].

Due to reactivity of GNPs (in contrast to “regular” gold), it is possible to modify the surface of these nanoparticles [23, 27]. Because of the high surface-to-volume ratio, even a slight surface modification leads to materials of different and interesting properties, that can be used *inter alia* in the design of sensors or biosensors [28–31]. For example, modification of GNPs was performed with a thiol derivative of 15-crown-5 [32]. Colloidal systems containing modified nanoparticles show selectivity towards biologically important potassium ions in water. Formation of sandwich-type complexes (2:1, L:M) leads to changes in distances between modified nanoparticles (aggregation), manifested by a change in color from red to blue. The same authors used thiol derivatives of 15-crown-5 (15-C-5) and 12-crown-4 (12-C-4) with different alkyl chain lengths to modify the surface of GNPs. In addition, carboxylic acid moieties were immobilized on the surface [33]. Systems modified with 15-crown-5 showed selectivity towards potassium ions. In the case of ethers with a smaller molecular cavity (12-C-4), a colorimetric response was obtained in the presence of crucial for living organisms sodium ions. A relationship between the length of the alkyl chain and the capability of ion recognition was found. The best results were obtained for nanoparticles modified with crown ethers having the shortest alkyl chains. This can be explained by interactions with neighboring carboxylic groups. Similar mechanism of potassium and sodium recognition was proposed for gold nanorods modified with 15-C-5 and 12-C-4 thiol derivatives [34]. Potassium selectivity (linear response for potassium 0.05–0.95 mM) was also found for nanogold modified with crown ether (15-C-5) dithiocarbamate [35]. Recently, aptamer-modified AuNPs have been proposed as a potassium selective colorimetric sensors [36, 37]. Depending on the recognition element properties nanomole range detection limits for potassium can be achieved. Earlier, comparable system, but based on nanosilver was also proposed for potassium determination in serum [38].

Above-mentioned examples of using nanosystems as simple colorimetric sensors for determination of ions of biological importance seem to be very attractive, especially comparing methods which needs more expensive or sophisticated equipment e.g., fluorescence [39], electrophoresis [40], atomic absorption spectrometry [41] or flame photometry [42, 43]—currently almost out of use in clinical analysis.

Nowadays nanosystems being not only easy to operate, but also enabling naked-eye detection of potassium can also be considered as a strong rival for potentiometric methods namely, ISE used both in clinical laboratories and in point of care testing [44–47].

Gold nanoparticles may be modified not only with macrocyclic compounds, but also with other compounds showing specific affinity to metal cations [30, 48–53], including dyes [54].

The objective of this work consists in the synthesis of new compounds and the preparation of novel materials that would be used for selective detection of cations of biological importance, i.e., potassium and sodium in water.

However, as derivatives facilitating binding the azobenzocrowns with metallic substrates are—to the authors’ best knowledge—not reported, attempts to obtain thiol derivatives of these compounds were made. To date, only one azobenzocrown derivative featuring sulfur atoms within the side chain was obtained, but the sulfur atoms were the part of thioether and thiophene moieties [55].

**Fig. 1:**
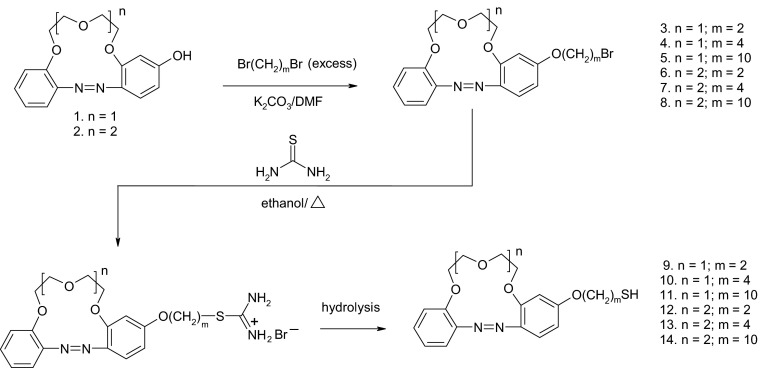
Synthesis of the thiol derivatives of azobenzocrowns

Derivatives of hydroxyazobenzocrowns with side chains containing thiol moieties were obtained (Fig. 1) and used for modification of GNPs. The properties of the plasmonic sensors, for potassium and sodium ions, based on the above mentioned compounds were investigated.

## Experimental

### General

**Fig. 2:**
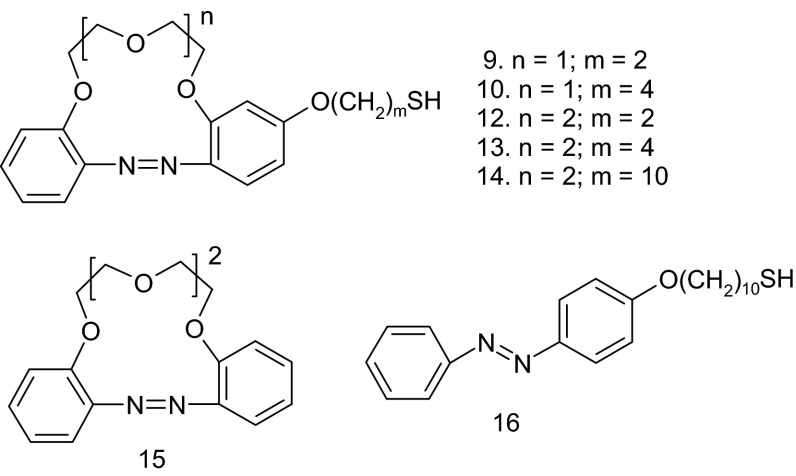
Compounds tested in plasmonic sensors

Unless otherwise stated, materials and solvents were of analytical reagent grade, obtained from commercial suppliers and used without further purification. 1,2-Dibromoethane, 1,4-dibromobutane, 1,10-dibromodecane, tetraethylenepentamine, tetrachloroauric acid trihydrate, lipoic acid, sodium citrate, and potassium citrate were obtained from Sigma-Aldrich. 4′-Hydroxyazobenzo-13-crown-4 (**1**) and 4′-hydroxyazobenzo-16-crown-5 (**2**) (Fig. 1) were prepared as previously described [56]. Compound **16** (Fig. 2) was synthesized as described in [57, 58].

Silica gel 60 (0.063–0.200 mm) (Merck) was used for column chromatography. Silica gel 60F_254_-coated glass plates (Merck) were used for preparative thin-layer chromatography. NMR spectra were recorded on a Varian instrument at 500 MHz. Chemical shifts are reported as δ [ppm] values in relation to TMS. Mass spectra were taken on a MALDISynapt G2-S HDMS (Waters Inc) mass spectrometer equipped with an electrospray ion source and q-TOF type mass analyzer. Mass spectra of the investigated compounds were recorded in the positive ion mode. FTIR spectra were recorded using a Mattson Genesis II instrument. UV–Vis spectra were recorded using a UNICAM UV 300 apparatus. Deionized water (18 MΩ cm, Hydrolab, Poland), subjected to further distillation, was used both for the preparation of AuNPs and all solutions used in the studies of their properties. All measurements were carried out at room temperature.

### Synthesis

#### Synthesis of bromo alkoxy derivatives of azobenzo-13-crown-4 and azobenzo-16-crown-5 (compounds **3**–**8**, Fig. 1)

A solution of 1,2-dibromoethane (3 mmol, 0.26 mL) or 1,4-dibromobutane (3 mmol, 0.35 mL) or 1,10-dibromodecane (3 mmol, 0.67 mL), compound **1** (0.6 mmol, 0.18 g) or compound **2** (0.6 mmol, 0.21 g), and potassium carbonate (6 mmol, 0.84 g) in dimethylformamide (DMF, 6 mL) was heated at 50 °C and stirred vigorously for 4 h. Next, the mixture was cooled down, diluted with water and extracted four times with a mixture of ethyl acetate and hexane (3:1, v/v). The red organic layer was washed with water, dried over anhydrous magnesium sulfate, filtered and evaporated in vacuo. The residue was dissolved in dichloromethane and immediately transferred onto the chromatographic column. The product was isolated using a mixture of methylene chloride and acetone (10:1, v/v) (compounds **3**–**5**) or a mixture of methylene chloride and acetone (10:1 and 5:1, v/v) (compounds **6**–**8**). The solvents were evaporated in vacuo. The obtained oily products undergo rapid en masse crystallization. Bromo derivatives (red–orange) were recrystallized from hexane, acetone-hexane or toluene-hexane.

*Compound***3** Yield 66 %. Mp 117–119 °C. ^1^H NMR (d-acetone), *trans* isomer, δ: 3.83 (2H, t, *J* = 6 Hz); 3.89 (4H, t, *J* = 4 Hz); 4.28–4.31 (4H, m); 4.49 (2H, t, *J* = 6 Hz); 6.79–6.81 (2H, m); 7.17 (1H, t, *J* = 8 Hz); 7.21 (1H, d, *J* = 8 Hz); 7.39 (1H, dt, *J*_1_ = 8 Hz, *J*_2_ = 2 Hz); 7.69 (1H, dd, *J*_1_ = 8 Hz, *J*_2_ = 2 Hz); 7.79 (1H, d, *J* = 9 Hz). ^13^C NMR (d-acetone), δ: 30.2; 68.5; 69.4; 69.7; 71.4; 72.0; 104.3; 108.3; 119.3; 122.3; 122.5; 125.8; 130.95; 138.9; 144.96; 153.8; 155.5; 161.5. FTIR (film): 3064; 2923; 2865; 1600; 1573; 1479; 1448; 1421; 1357; 1282; 1244; 1191; 1171; 1118; 1051; 933; 897; 758; 736 cm^−1^. HRMS (ESI): [M + H]^+^ = 407.0599 calculated for C_18_H_20_N_2_O_4_Br 407.0606.

*Compound***4** Yield 83 %. Mp 88–90 °C. ^1^H NMR (d-acetone), *trans* isomer, δ: 1.98 (2H, quintet, *J* = 7 Hz); 2.09 (2H, quintet, *J* = 7 Hz); 3.63 (2H, t, *J* = 7 Hz); 3.89 (4H, t, *J* = 4 Hz); 4.18 (2H, t, *J* = 6 Hz); 4.28–4.31 (4H, m); 6.77–6.78 (2H, m); 7.17 (1H, t, *J* = 8 Hz); 7.21 (1H, d, *J* = 8 Hz); 7.38 (1H, t, *J* = 8 Hz); 7.69 (1H, d, *J* = 8 Hz); 7.78 (1H, d, *J* = 9 Hz). ^13^C NMR (d-acetone), δ: 27.9; 33.8; 67.5; 69.4; 69.7; 71.4; 72.0; 104.1; 108.3; 119.2; 122.4; 122.5; 125.6; 130.8; 138.6; 145.0; 153.7; 155.6; 162.4. FTIR (film): 3065; 2927; 2870; 1600; 1571; 1492; 1445; 1290; 1246; 1190; 1127; 1046; 934; 875; 840; 757 cm^−1^. HRMS (ESI): [M + H]^+^ = 435.0914 calculated for C_20_H_24_N_2_O_4_Br 435.0919.

*Compound***5** Yield 71 %. Mp 81–83 °C. ^1^H NMR (d-acetone), *trans* isomer, δ: 1.3–1.57 (8H, m); 1.8–1.9 (8H, m); 3.52 (2H, t, *J* = 7 Hz); 3.98–4.0 (4H, m); 4.14 (2H, t, *J* = 7 Hz); 4.32 (4H, t, *J* = 4 Hz); 6.75–6.79 (2H, m); 7.18 (1H, t, *J* = 8 Hz); 7.22 (1H, dd, *J*_1_ = 8 Hz, *J*_2_ = 1 Hz); 7.39 (1H, dt, *J*_1_ = 8 Hz, *J*_2_ = 2 Hz); 7.69 (1H, dd, *J*_1_ = 8 Hz, *J*_2_ = 2 Hz); 7.79 (1H, d, *J* = 8 Hz). ^13^C NMR (d-acetone), δ: 28.8; 28.9; 33.0; 34.2; 68.4; 69.4; 69.7; 71.4; 71.95; 104.0; 108.3; 119.2; 122.42; 122.44; 125.6; 130.8; 138.5; 145.0; 153.6; 155.6; 162.6. FTIR (Nujol): 1596; 1280; 1237; 1172; 1046; 1011; 896; 824; 748; 723 cm^−1^. HMRS (ESI): [M + H]^+^ = 519.1856 calculated for C_26_H_36_N_2_O_4_Br 519.1858.

*Compound***6** Yield 70 %. Mp 131–133 °C. ^1^H NMR (d-acetone), *trans* isomer, δ: 3.66 (4H, s); 3.83 (2H, t, *J* = 5 Hz); 3.86–3.92 (4H, m); 4.21–4.25 (2H, m); 4.28–4.31 (2H, m); 4.50 (2H, t, *J* = 5 Hz); 6.72 (1H, dd, *J*_1_ = 9 Hz, *J*_2_ = 2 Hz); 6.80 (1H, d, *J* = 2 Hz); 7.08 (1H, t, *J* = 8 Hz); 7.20 (1H, d, *J* = 8 Hz); 7.37 (1H, t, *J* = 8 Hz); 7.57 (1H, d, *J* = 8 Hz); 7.72 (1H, d, *J* = 9 Hz). ^13^C NMR (d-acetone), δ: 30.2; 68.5; 69.4; 69.5; 69.6; 69.7; 70.5; 70.8; 101.5; 106.7; 115.1; 120.9; 123.1; 124.8; 130.6; 138.1; 144.1; 152.8; 155.5; 161.6. FTIR (film): 3048; 2930; 2874; 1600; 1573; 1489; 1454; 1355; 1266; 1189; 1117; 1054; 938; 837; 735 cm^−1^. HRMS (ESI): [M + Na]^+^ = 473.0681 calculated for C_20_H_23_N_2_O_5_BrNa 473.0688.

*Compound***7** Yield 82 %. Mp 85–87 °C. ^1^H NMR (d-acetone), *trans* isomer, δ: 1.86–1.94 (2H, m); 2.00 (2H, quintet, *J* = 7 Hz); 3.62 (2H, t, *J* = 7 Hz); 3.65–3.75 (4H, m); 3.88–3.95 (4H, m); 4.15–4.20 (2H, m); 4.22–4.30 (4H, m); 6.65–6.72 (2H, m); 7.13 (1H, t, *J* = 8 Hz); 7.19 (1H, d, *J* = 8 Hz); 7.36 (1H, t, *J* = 8 Hz); 7.56 (1H, d, *J* = 8 Hz); 7.70 (1H, d, *J* = 9 Hz). ^13^C NMR (d-acetone), δ: 27.9; 33.9; 67.5; 69.4; 69.5; 69.7; 70.5; 70.8; 101.2; 106.6; 115.1; 120.9; 123.1; 124.9; 130.4; 137.8; 144.1; 152.8; 155.5; 162.4. FTIR (film) 3065; 2926; 2874; 1600; 1501; 1445; 1290; 1253; 1187; 1115; 1047; 937; 836; 756 cm^−1^. HRMS (ESI): [M + Na]^+^ = 501.1002 calculated for C_22_H_27_N_2_O_5_NaBr 501.1001.

*Compound***8** Yield 73 %. Mp 76–77 °C. ^1^H NMR (d-acetone), *trans* isomer, δ: 1.26–1.50 (12H, m); 1.82–1.92 (4H, m); 3.53 (2H, t, *J* = 6.8 Hz); 3.67–3.74 (4H, m); 3.90–3.95 (4H, m); 4.15 (2H, t, *J* = 7 Hz); 4.25 (2H, t, *J* = 5 Hz); 4.28 (2H, t, *J* = 4 Hz); 6.71 (1H, dt, *J*_1_ = 9 Hz, *J*_2_ = 2 Hz); 6.73 (1H, d, *J* = 2 Hz); 7.10 (1H, t, *J* = 8 Hz); 7.20 (1H, d, *J* = 8 Hz); 7.38 (1H, dt, *J*_1_ = 2 Hz, *J*_2_ = 8 Hz); 7.57 (1H, dd, *J*_1_ = 8 Hz, *J*_2_ = 2 Hz); 7.72 (1H, d, *J* = 9 Hz). ^13^C NMR (d-acetone), δ: 26.1; 28.1; 33.0; 34.2; 68.4; 69.4; 69.5; 69.6; 70.5; 70.7; 101.2; 106.6; 115.1; 120.9; 123.1; 125.0; 130.4; 137.6; 144.1; 152.8; 155.5; 162.7. FTIR (film): 3065; 3041; 2926; 2854; 1601; 1488; 1442; 1290; 1259; 1188; 1133; 1115; 1049; 938; 737 cm^−1^. HRMS (ESI): [M + Na]^+^ = 585.1933 calculated for C_28_H_39_N_2_O_5_NaBr 585.1940.

#### Synthesis of thiol derivatives of azobenzo-13-crown-4 and azobenzo-16-crown-5 (compounds **9**–**14**, Fig. 1)

The respective bromo derivative **3**–**8** (0.3 mmol) and thiourea (0.4 mmol, 30.4 mg) in degassed ethanol (99.8 %, 10 mL) were stirred and heated at reflux for 5–6 h under argon.

The hydrolysis of the above intermediate product was carried out using two methods:

*Method A* the reaction mixture was treated with aqueous LiOH solution (2 mL, 0.3 M) and the stirring mixture was heated at 60 °C for 1 h.

*Method B* to the reaction mixture water (0.1 mL) and tetraethylenepentamine (0.08 mmol, 0.015 mL) were added and the mixture was heated for 1.5 h at 40–50 °C.

Slightly better results were obtained with method A.

The mixture after hydrolysis was extracted three times with chloroform. The organic layer was washed with water, dried over anhydrous magnesium sulfate, filtered and evaporated in vacuo. The residue was treated with acetone (ca. 15 mL), stirred and filtered. The filtrate was evaporated and dissolved in a small amount of dichloromethane. The solution was applied onto preparative TLC plates. The plates were developed in dichloromethane-methanol (15:1, v/v). The product was washed off the silica gel using a dichloromethane-methanol (10:1, v/v) mixture. The silica gel was filtered and the filtrate was evaporated in vacuo. The residue was treated with a small amount of dichloromethane and filtered again. After solvent evaporation an oily, yellow–orange product was obtained.

The thiol derivatives were stored in tightly closed small vials at a reduced temperature (+4 °C).

*Compound***9** Yield 27 %. ^1^H NMR (d-benzene), mixture of *trans* and *cis* isomers, δ: 2.60–2.67 (2H, m); 3.55–4.0 (10H, m); 5.91 (0.33H, dd, *J*_1_ = 9 Hz, *J*_2_ = 2 Hz); 6.35–6.43 (1.33H, m); 6.48–6.52 (0.67H, m); 6.61 (0.33H, t, *J* = 7 Hz); 6.74–6.92 (2H, m); 6.96–7.02 (1H, m); 7.99–8.08 (1.34H, m). FTIR (film): 3063; 2924; 2866; 2279; 1599; 1478; 1447; 1285; 1249; 1172; 1129; 1049; 934; 876; 841; 813; 757 cm^−1^. HRMS (ESI): [M + Na]^+^ = 383.1038 calculated for C_18_H_20_N_2_O_4_NaS 383.1041; [M’(disulfide) + Na]^+^ = 741.2014 calculated for C_36_H_38_N_4_O_8_NaS_2_ 741.2029.

*Compound***10** Yield 38 %. ^1^H NMR (d-benzene), mixture of isomers, δ: 1.50–1.60 (2H, m); 1.65–1.72 (2H, m); 2.47 (2H, q, *J* = 7 Hz); 3.43–3.50 (2H, m); 3.60–3.68 (4H, m); 3.90–3.97 (2H, m); 3.99–4.05 (2H, m); 5.96 (0.35H, dd, *J*_1_ = 9 Hz, *J*_2_ = 2 Hz); 6.36–6.40 (0.65H, m); 6.46 (0.65H, dd, *J*_1_ = 9 Hz, *J*_2_ = 2 Hz); 6.49–6.54 (1H, m); 6.62 (0.35H, t, *J* = 8 Hz); 6.77–6.81 (1H, m); 6.87–6.93 (1H, m); 7.00 (0.65H, t, *J* = 8 Hz); 7.93–7.98 (0.65H, m); 8.00–8.03 (0.65H, m). ^13^C NMR (d-benzene), mixture of isomers, δ: 25.9; 28.0; 30.0; 30.1; 38.5; 67.1; 67.5; 68.5; 69.4; 69.6; 69.7; 71.4; 71.6; 71.8; 72.9; 102.9; 104.5; 104.8; 107.9; 114.2; 118.4; 120.5; 121.0; 121.9; 122.2; 123.7; 125.7; 128.5; 130.5; 138.9; 139.5; 144.9; 145.2; 147.5; 152.8; 153.6; 156.0; 159.5; 162.2. FTIR (film): 3065; 2925; 2867; 1600; 1572; 1494; 1476; 1447; 1289; 1249; 1189; 1115; 1046; 934; 876; 825 cm^−1^. HRMS (ESI): [M’ + Na]^+^ = 797.2636 calculated for C_40_H_46_N_4_O_8_NaS_2_ 797.2655.

*Compound***11** Yield 32 %. ^1^H NMR (d-acetone), δ: 1.30–1.48 (8H, m); 1.49–1.58 (2H, m); 1.66–1.78 (2H, m); 1.78–1.88 (2H, m); 2.75 (2H, t, *J* = 7 Hz); 3.32–3.36 (2H, m); 3.60–3.66 (1H, m); 3.86–4.00 (4H, m); 4.11 (2H, t, *J* = 6 Hz); 4.26–4.33 (4H, m); 6.37 (1H, d, *J* = 2 Hz); 6.74 (1H, dd, *J*_1_ = 9 Hz, *J*_2_ = 2 Hz), 7.14–7.22 (2H, m), 7.37–7.40 (1H, m), 7.66 (1H, d, *J* = 8 Hz), 7.75 (1H, d, *J* = 9 Hz). FTIR (film): 3063; 2927; 2854; 2246; 1601; 1572; 1456; 1289; 1244; 1190; 1119; 1039; 931; 901; 823; 756; 736 cm^−1^. HRMS (ESI): [M’ + Na]^+^ = 965.4521 calculated for C_52_H_70_N_4_O_8_NaS_2_ 965.4533.

*Compound***12** Yield 37 %. ^1^H NMR (d-benzene), mixture of isomers, δ: 2.57–2.66 (2H, m); 3.48–3.63 (10H, m); 3.80–3.87 (4H, m); 5.86 (0.35 H, dd, *J*_1_ = 9 Hz, *J*_2_ = 2 Hz); 6.29–6.37 (1.35H, m); 6.46 (0.65H, d, *J* = 2 Hz); 6.58–6.66 (1.35H, m); 6.75–6.88 (1H, m); 6.96–7.10 (1H, m); 7.79–7.85 (0.65H, m); 7.91–7.95 (0.65H, m). FTIR (film): 3064; 2923; 2868; 2267; 1598; 1486; 1446; 1284; 1249; 1188; 1116; 1052; 930; 839; 754 cm^−1^. HRMS (ESI): [M’ + Na]^+^ = 829.2517 calculated for C_40_H_46_N_4_O_10_NaS_2_ 829.2553.

*Compound***13** Yield 46 %. ^1^H NMR (d-acetone), δ: 1.50–1.90 (4H, m); 2.74 (2H, q, *J* = 7 Hz); 3.65–3.95 (8H, m); 4.10–4.30 (6H, m); 6.26 (~0.5H, dd, *J*_1_ = 9 Hz, *J*_2_ = 2 Hz); 6.52–6.57 (~1H, m); 6.66–6.73 (~1H, m); 6.87–6.94 (~1H, m); 7.04–7.21 (~2H, m); 7.34–7.40 (~1H, m); 7.56 (~0.5H, d, *J* = 8 Hz); 7.71 (~0.5H, d, *J* = 9 Hz). FTIR (film): 3067; 2927; 2875; 2459; 1600; 1570; 1488; 1444; 1291; 1253; 1188; 1116; 1048; 939; 824; 753; 659 cm^−1^. HRMS (ESI): [M’ + Na]^+^ = 885.3169 calculated for C_44_H_54_N_4_O_10_NaS_2_ 885.3179.

*Compound***14** Yield 43 %. ^1^H NMR (d-benzene), δ: 1.20–1.45 (12H, m); 1.64–1.76 (4H, m); 2.61 (2H, t, *J* = 7 Hz); 3.60–3.75 (10H, m); 3.94 (2H, t, *J* = 4 Hz); 3.99 (2H, t, *J* = 4 Hz); 6.53 (1H, dd, *J*_*1*_ = 9 Hz, *J*_*2*_ = 2 Hz); 6.62 (1H, d, *J* = 2 Hz); 6.70–6.72 (1H, m); 6.94 (1H, t, *J* = 8 Hz); 7.08 (1H, t, *J* = 7 Hz); 7.92 (1H, d, *J* = 8 Hz); 8.10 (1H, d, *J* = 9 Hz). FTIR (film): 3065; 2926; 2854; 2607; 1600; 1496; 1445; 1354; 1290; 1250; 1189; 1115; 1053; 931; 833; 753 cm^−1^. HRMS (ESI): [M’ + Na]^+^ = 1053.5043 calculated for C_56_H_78_N_4_O_10_NaS_2_ 1053.5057.

### Preparation of colloidal gold nanoparticles (AuNPs)

The synthesis of colloidal spherical GNPs sized ca. 20 nm was carried out by the method involving the reduction of tetrachloroauric acid (HAuCl_4_) with citrates. The procedure had been earlier described in the literature [32]. AuNPs were prepared in two variants. Depending on macrocycle size, sodium—for 16-membered thiol azobenzocrowns [**Au1**] or potassium for 13-membered macrocycles [**Au2**]—citrates were used for nanogold preparation.

An aqueous solution of HAuCl_4_ (180 mL, 1.6 mM) was heated to the boiling point. An aqueous solution of sodium or potassium citrate (18 mL, 38.8 mM) was added in one portion upon vigorous stirring. The solution changed color from light yellow to dark blue after ca. 20 s and to burgundy after another minute. The solution was heated for another 10 min. Then, the flask was transferred onto a room temperature surface and stirred for another 15 min. Colloidal gold was stored without the access of light at a reduced temperature (10–15 °C).

### Modification of colloidal gold nanoparticles with thiol derivatives of 16- and 13-membered azobenzocrowns

A solution obtained by mixing AuNPs colloidal solution (5 mL) and water (10 mL) was treated with NaOH (ca. 0.06 mL (2 drops), 0.25 M) for pH adjustment (7–8). The flask was then placed on a magnetic stirrer set to mild stirring. Ca. 0.06 mL (2 drops) of an ethanolic solution of the thiol azobenzocrown derivative prepared by dissolving 5 mg of the compound in ethanol (0.5 mL) was added upon stirring to the GNPs sol. The mixture was stirred for 48 h at room temperature, protected from light. The modified GNPs were stored without the access of light at a reduced temperature (10–15 °C).

### Preparation of bifunctionalized gold nanoparticles

#### Modification of nanogold using lipoic acid (Tioctic Acid, TA)

The procedure was carried out analogously to that described in the literature [33, 59]. AuNPs solution (25 mL) was filtered through a glass fiber filter and transferred into a round-bottom flask equipped with a stirrer. Next, to the flask water (25 mL) and NaOH (0.6 mL of 0.25 M for **Au1** preparation) or KOH (0.6 mL of 0.25 M for **Au2** preparation) for pH adjustment (ca. 11) were added. The solutions were treated with TA (1.6 mg). The resulting mixture was carefully stirred for 24 h at room temperature, protected from light. **Au1.TA** and **Au2.TA** were obtained. The resulting modified colloidal gold solutions were stored without the access of light at room temperature.

#### Modification of gold nanoparticles coated with lipoic acid with thiol derivatives of azobenzocrowns

A sample of **Au.TA** (5 mL) was placed in a 25 mL round-bottom flask equipped with a stirrer. To this NaOH (ca. 0.03 mL (1 drop), 0.25 M, in case of **Au1.TA**) or KOH (in case of **Au2.TA**) was added to adjust the pH (7–8). The flask was placed on a magnetic stirrer set to mild stirring. To the stirred GNPs an ethanolic solution (0.06 mL, 2 drops) of thiol azobenzocrown (5 mg in 0.5 mL of ethanol) was added. The mixture was stirred for 48 h at room temperature, protected from light. AuNPs with anchored TA and thiol azobenzocrown derivatives were extracted with diethyl ether to remove the unbound thiol derivative of azobenzocrown. The resulting modified nanogold colloid was stored without the access of light at room temperature.

### Spectrophotometric measurements

#### UV–Vis spectroscopy

In typical spectrophotometric titration procedure, a portion of salt solution was introduced into the cuvette [poly(methyl methacrylate), optical path length 1 cm] with GNPs solution at appropriate concentration. The spectrum was recorded. Another portion of salt solution was added. The contents of the cuvette typically were stirred for 1 min after each addition of salt solution. Linear response of the nanogold system towards potassium cations was estimated from titration experiments as relationship (A_0_ − A) = f (c_KCl_) or (A − A_0_) = f (c_KCl_), where A is absorbance upon addition of salt and A_0_ is absorbance nanogold before salt addition. Different wavelengths were checked, including among the others the wavelength value at which the difference between absorbance of nanogold and absorbance of system upon salt addition was the highest.

#### Preparation of nanogold samples for infrared spectroscopy

The samples of colloidal gold solution were centrifuged (10 min 13,000 rpm). Next, the colorless supernatant was decanted, and the tube was filled with water and centrifuged again (10 min, 13,000 rpm). Water was decanted and the precipitate was left to dry at room temperature and later for 2 h at 40 °C. The precipitate was mixed with KBr and compressed into pellets.

## Results and discussion

### Synthesis of thiol derivatives of azobenzocrown ethers

4′-Hydroxyazobenzocrowns which can be efficiently prepared from azoxybenzocrowns in a reaction analogous to the Wallach rearrangement [56, 60, 61] were selected as substrates for the synthesis of thiol derivatives. Azobenzocrowns with peripheral hydroxyl groups are universal substrates for further modifications. They may be used as starting compounds in the synthesis of, e.g., lariat azobenzocrowns and bisazobenzocrowns [56, 62, 63].

At first, the bromo derivatives were synthesized (Fig. 1) by reaction of 4′-hydroxyazobenzocrowns with an excess of 1,2-dibromoethane, 1,4-dibromobutane, or 1,10-dibromodecane in the presence of potassium carbonate in DMF. The reactions were carried out at 50 °C for 4 h. Attempts to obtain bromo derivatives in reactions carried out in acetone in the presence of potassium carbonate (reflux, 24 h) as well as in alcohols in the presence of hydroxides (for example, KOH, ethanol, reflux, 5 h) were also made. The highest yields (up to 80 %) were obtained for reactions carried out in DMF, while the lowest yields were obtained for reactions carried out in alcoholic environments (less than 30 %). The resulting crude, reactive bromo derivatives of azobenzocrowns require a special treatment. Purification and crystallization have to be performed immediately after the synthesis, but pure, crystalline product can be stored at room temperature for as long as several months.

The synthesis of thiol derivatives of azobenzocrowns posed much difficulty due to the azo group’s susceptibility to redox reactions as well as to the sensitivity of azobenzocrowns (and particularly their *cis* isomers) to acidic environment. Methods that have proved to be efficient in the synthesis of azobenzene derivatives were taken into account when searching for the method for the synthesis of thiol derivatives of azobenzocrowns. Three methods were tested. The first method involved the reaction of bromo derivatives with thiourea followed by basic hydrolysis of the resulting isothiouronium salt [57, 58]. The second, involved the reaction of bromo derivatives with sodium thiosulfate followed by acidic hydrolysis of the resulting Bunte salt [64]. Unfortunately, this method failed at the stage of Bunte salt hydrolysis. The third way was the reaction of mesyl derivatives with potassium thioacetate followed by basic hydrolysis of the resulting thioacetate derivative [65]. This method allowed us to obtain the desired final product, albeit with a low yield. Problems were encountered already at the stage of the synthesis of the mesyl derivative, being associated mostly with the stability of that derivative, as well as at the two final stages of the reaction with potassium thioacetate and the hydrolysis of thioacetate derivative. A significant contribution of ether bond cleavage and recovery of the starting 4′-hydroxyazobenzocrown was observed. The problem was also observed, however to a lesser extent, in reactions wherein thiourea was used (the first method). Thus the first method (of greater simplicity than third way) was chosen as a way to obtain the product in relatively highest yields.

Figure 1 presents the scheme for preparation of the thiol derivatives of azobenzocrowns by the thiourea method. The first stage was carried out under typical conditions, i.e., 5–6 h at ethanol’s boiling point under inert gas atmosphere. The hydrolysis conditions required modifications particularly in the case of compounds **9** and **12** featuring the shortest oxyethylene linkers. Typically, the hydrolysis was carried out in an ethanol–water solutions in the presence of lithium hydroxide at 60 °C and in the presence of tetraethylenepentamine at 40–50 °C. The yields of the syntheses of thiol azobenzocrown derivatives from the respective bromo derivatives were in the range of 27–46 %.

Similar to other derivatives of azobenzocrowns, the thiol derivatives exist as two isomers, *cis* and *trans,* with *cis* isomers being more stable than these of azobenzene derivatives. Unfortunately, attempts to crystallize these compounds in pure isomeric forms had failed, and only oily mixtures of both isomers, with *trans* isomer being the predominant one, were available. The compounds were characterized by means of ^1^H NMR spectrometry immediately after preparation. They were stored at low temperatures. However, significant oxidation into disulfides could not be prevented and it was reflected in the obtained mass spectra (ES+). The mixtures of thiol compounds and disulfides featured five different geometrical isomers (which can be distinguished e.g. using TLC). This fact, made spectral analyses more difficult. Thus, only an example ^13^C NMR spectrum of thiol compound is presented here and the spectral analyses are limited mostly to ^1^H NMR, FTIR and MS spectra.

### The use of thiol azobenzocrown derivatives for modification of gold nanoparticles

Azobenzocrowns have proven to be useful as ionophores for potentiometric sensors for sodium and potassium ions, depending on the macroring size [10, 11, 56]. The selectivity of ISEs with membranes modified with lipophilic azobenzocrown derivatives was found to be associated with formation of sandwich-type complexes (2:1, L:M, similarly as sandwich-type complexes are formed in a solid state by *trans* isomers of azobenzocrowns [66, 67].

One may notice the similarity to formation of sandwich-type complexes by crown ethers, which had been previously used for modification of GNPs [32]. The results of these studies suggest that the behavior of ionophores anchored to nanogold particles is very likely analogous to that observed in the ion selective electrodes. Therefore, we assumed that the proposed ligands constitute a promising group of compounds that may be used for modification of GNPs.

The obtained thiol derivatives of azobenzocrowns were anchored to the surfaces of GNPs. The presence of disulfide derivatives detected in the samples of thiol azobenzocrowns was found not to have an adverse impact on the chemisorptions of compounds onto nanogold. It could be explained by the fact that disulfides upon S–S bond cleavage drive chemisorptions onto GNPs what also leads to the formation of a self-assembled monolayers. Dissociation is clearly favored for the disulfide with subsequent formation of strongly bound thiolates [68].

We are presenting the preliminary results of one of our research directions aimed at assessing the potential of azobenzocrowns for being used in the development of plasmonic sensors based on colloidal GNPs and sensitive towards alkali metal ions.

Since the derivatives of the 16-membered azobenzocrown forming a sandwich-type complex with potassium ions are characterized by selectivity towards potassium ions as compared to sodium ions when used as ionophores in ISE membranes, similar results may be expected for plasmonic sensors featuring compounds of this type. Analogously, selectivity towards sodium ions may be expected for sensors containing 13-membered azobenzocrowns.

Colloidal GNPs were obtained by reduction of tetrachloroauric acid with sodium citrate (**Au1**) or potassium citrate (**Au2**) in aqueous solutions. The reagent ratio was selected so that the size of nanoparticles was ca. 20 nm and the colloid was burgundy in color. **Au1** was used for modification of gold with 16-membered azobenzocrown derivatives, while **Au2** was used for modification of gold with 13-membered azobenzocrown derivatives. Modification of GNPs with only the thiol derivatives of azobenzocrowns afforded relatively unstable colloidal systems with stability the poorer the shorter the peripheral hydrocarbon chains. Therefore, bifunctionalized GNPs containing lipoic acid and thiol azobenzocrown derivatives were the main objects of this study. The systems were characterized by high stability and capability of being stored at room temperature for several months.

Two variants for preparation of bifunctionalized nanogold were initially tested. In the first variant, samples of nanogold modified with lipoic acid were centrifuged. Centrifuged nanogold was frequently less stable than its non-centrifuged counterpart. In the second variant (described herein), which was studied more extensively, centrifugation was abandoned and the workup was limited to extraction following the second stage of modification, i.e., the anchoring of the thiol crown ether, so as to remove non-nanogold-bound azobenzocrowns. All samples prepared in line with the second variant were characterized by high stability regardless of the concentration of GNPs.

Spectrophotometric studies were performed for gold colloids modified with the thiol azobenzocrowns as well as for the gold colloidal solution containing azobenzocrown **15** and for nanogold with thiol derivative of azobenzene not including a macrocyclic ring **16** which were used for comparative reasons (Fig. 2).

**Fig. 3:**
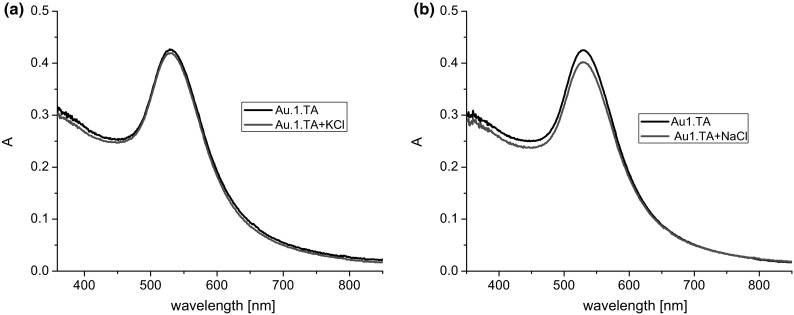
UV–Vis spectra of **Au1.TA** (2.4 mL, 0.085 mM) upon addition of **a** potassium chloride (1.5 mL, 0.05 M), **b** sodium chloride (1.5 mL, 0.05 M)

The properties of colloidal gold samples were analyzed using UV–Vis spectroscopy. Studies included conventional spectrophotometric titration, i.e., registering spectra of freshly prepared mixtures of nanogold sol and various quantities of salts (KCl, NaCl), and examination of the effect of the presence of Ca^2+^ and Mg^2+^ ions on the absorption spectra. For comparative reasons, in order to confirm that non-modified nanogold, nanogold modified with lipoic acid (**AuTA**), nanogold modified by non-cyclic compound **16**, and nanogold in the presence of unbound macrocycle **15** did not interact with K^+^ and Na^+^ ions within a certain range of concentrations of these ions, the respective UV–Vis spectra were recorded. Figure 3 exemplifies spectra of nanogold modified with lipoic acid recorded in the presence of potassium or sodium ions at concentrations of ca. 0.02 M. No shift was observed in the absorption maximum at λ = 528 nm, characteristic for nanoparticles having the diameter of ca. 20 nm [32]. However, one should mention that nanogold prepared by this method (as well as the non-modified colloidal gold) interacts with K^+^ or Na^+^ ions when the concentration of ions within the colloidal gold solution is ca. 0.04 M or more, leading to a change in the color from burgundy to purplish blue.

**Fig. 4:**
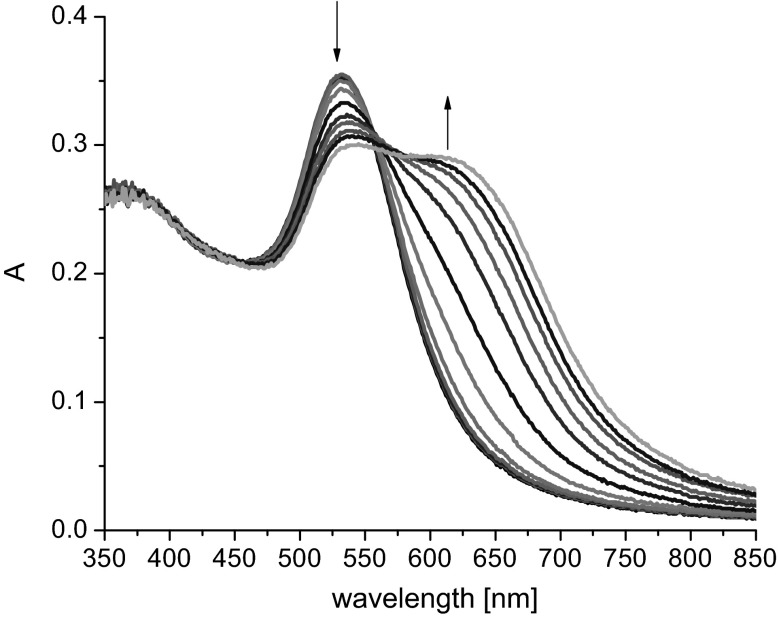
Changes in UV–Vis spectrum of **Au1.TA.12** (2.2 mL, 0.022 mM) upon titration with KCl solution (0.05 M, titration step: 0.1 mL)

The effect of the initial concentration of modified nanogold particles on spectral changes caused by the addition of potassium salt was studied with nanogold bifunctionalized with lipoic acid and compound **12** (**Au1.TA.12)**. In the case of nanogold at a concentration of 0.022 mM, the first spectral changes were observed at potassium level 7.7 mM; and the respective potassium levels for nanogold concentrations of 0.095 and 0.36 mM were 8.2 and 12 mM, respectively. Bathochromic shifts (20–30 nm) of the absorption band characteristic for nanogold and a new band shifted about 100 nm upon addition of KCl solution were observed. It was noticed that the lower nanogold concentration the more distinct is the new band. Figure 4 presents the spectral changes observed upon spectrophotometric titration of **Au1.TA.12** of the lowest among all studied so far concentrations (0.022 mM) with KCl.

**Fig. 5:**
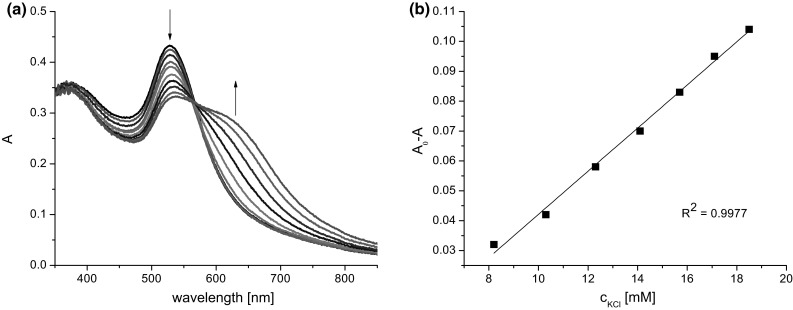
**a** Changes in UV–Vis spectrum of **Au1.TA.12** (2.3 mL, 0.095 mM) upon titration with KCl solution (0.05 M, titration step: 0.15 mL), **b** relationship between absorbance and potassium chloride concentration (8.2–18 mM) given as a difference |A_0_ – A|, where A is absorbance upon addition of salt and A_0_ is absorbance of **Au1.TA.12** before salt addition, at 528 nm

Figure 5a presents the spectral changes observed upon titration of **Au1.TA.12** of four times higher concentration than above (0.095 mM) with KCl. In Fig. 5b the relationship between concentration of KCl (8.2–18 mM) and changes in absorbance, given as a difference A_0_ − A, where A is absorbance upon addition of salt and A_0_ is absorbance of **Au1.TA.12** before salt addition, at 528 nm is shown. At wavelengths over 600 nm a narrower range of linear response (10–18 mM) was observed.

**Fig. 6:**
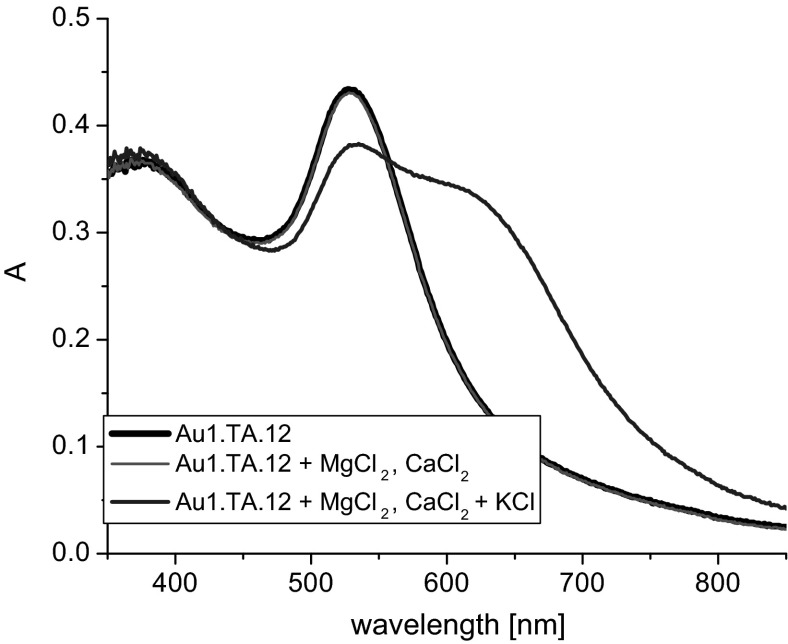
Comparison of UV–Vis spectra of **Au1.TA.12** (2.3 mL, 0.095 mM) registered upon addition of mixture of magnesium and calcium chlorides (0.3 mL, 8 × 10^−4^ M) and the effect of addition of potassium chloride solution (0.6 mL, 0.05 M)

The effect of the presence of Ca^2+^ and Mg^2+^ ions on the position and shape of the absorption band of modified nanogold was also examined. To a sol of **Au1.TA.12** a mixture of salts: CaCl_2_ and MgCl_2_ was added. No changes in absorption spectra were observed at following concentrations of salts: Ca^2+^ (9.2·10^−5^ M) and Mg^2+^ (9.2·10^−5^ M) (Fig. 6); however, the concentration levels of these ions have to be appropriately low. (Addition of potassium chloride (0.6 mL, 0.05 M) generates spectral changes shown with the blue line in Fig. 6). When the total concentration of calcium and magnesium ions exceeds 4.3 × 10^−4^ M, the color of the nanogold changes from burgundy to purplish blue (also in the case of unmodified colloidal gold).

**Fig. 7:**
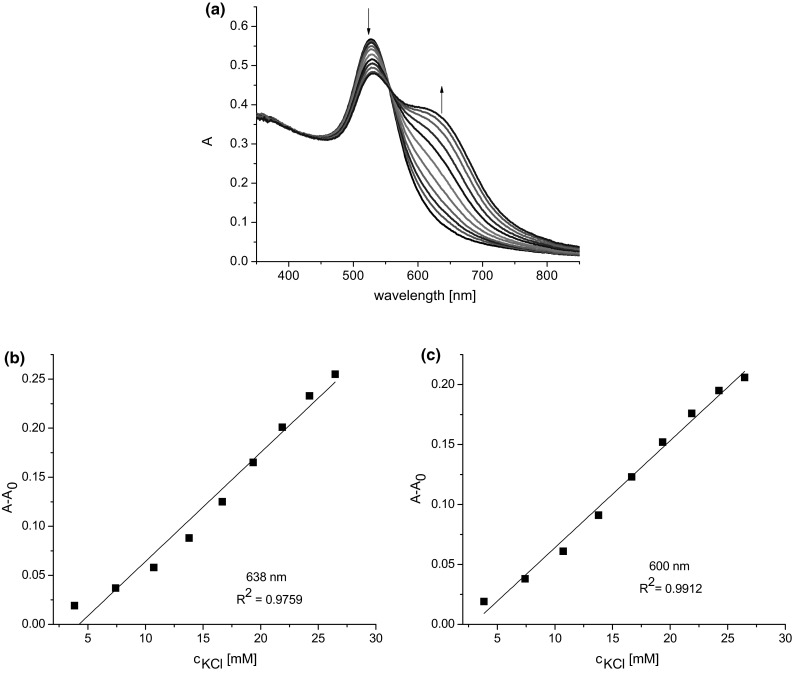
**a** Changes in UV–Vis spectra of **Au1.13** (2.5 mL, 0.100 mM) upon titration with potassium chloride (0.1 M; titration step 0.1 mL) and the spectrophotometric response of **Au1.13** towards potassium chloride at: **b** 638 nm, **c** 600 nm

Figure 7a presents spectral changes observed for colloidal gold modified with crown **13** upon its titration with potassium chloride. At a nanogold concentration of 0.1 mM the first evident spectral change was observed at a KCl concentration of 3.8 mM. Comparison of the range of linear spectral response towards potassium salt (3.8–26 mM) at 600 nm and at 638 nm (at this wavelength the difference between absorbance of nanogold and absorbance of system upon salt addition is the highest) is shown in Fig. 7b, c. From this comparison, 600 nm seems to be a better choice as an analytical wavelength for potassium determination.

**Fig. 8:**
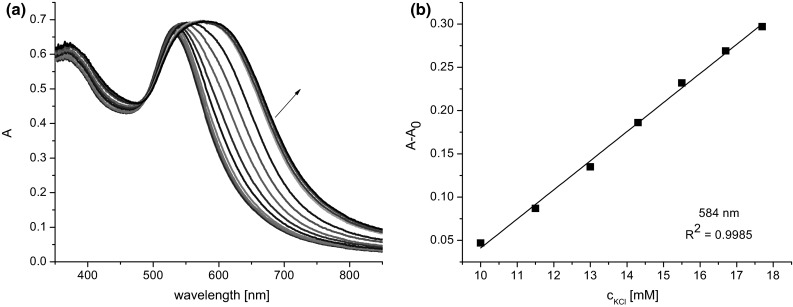
**a** Changes in UV–Vis spectrum of **Au1.TA.13** (2.0 mL, 0.25 mM) upon titration with KCl solution (0.05 M, titration step: 0.1 mL), **b** linear range of relationship between absorbance and potassium chloride concentration (10–17.7 mM) given as a difference A − A_0_, where A is absorbance upon addition of salt and A_0_ is absorbance of **Au1.TA.13** before salt addition, at 584 nm

Figure 8a presents, for comparative purposes, spectral changes recorded upon titration of **Au.TA.13** colloid at a higher concentration (0.25 mM) with potassium chloride solution. The concentration of GNPs has a high impact on the shape of the absorption band. The relationship between changes of absorbance and potassium chloride concentration (in this case for 584 nm) is also shown (Fig. 8b).

Compared to **Au1.TA.12**, the spectral changes observed upon titration of **Au1.TA.13** (containing a 16-membered azobenzocrown with oxybutylene linker) with potassium salt were more time-stable.

The spectra of colloidal gold modified with azobenzocrown **14** (with the longest chain) and colloidal gold modified both with azobenzocrown **14** and lipoic acid were compared. No significant spectral differences were observed. The concentration of K^+^ ions at which the first evident spectral change was observed was 8.3 mM for **Au1.TA.14** and 9 mM for **Au1.14** at nanogold concentration 0.27 mM.

**Fig. 9:**
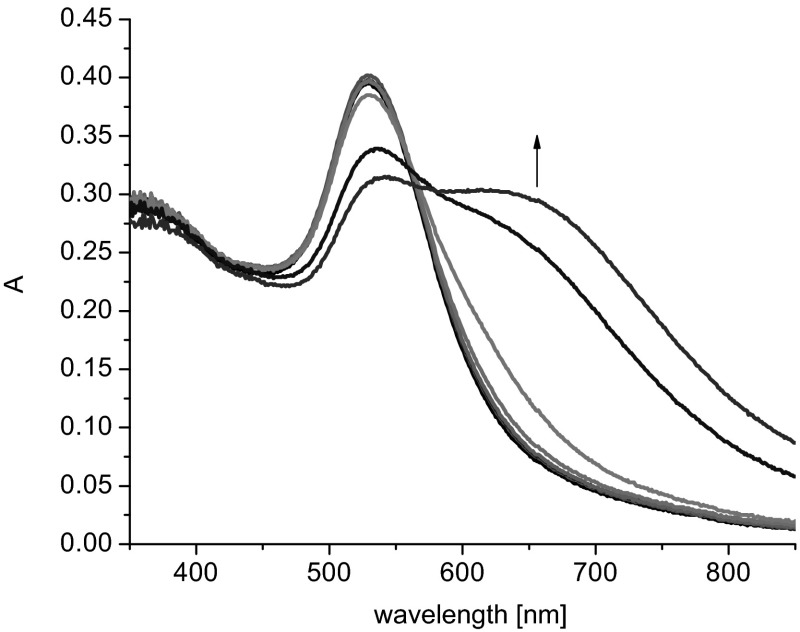
Changes in UV–Vis spectrum of **Au2.TA.10** (2.3 mL, 0.079 mM) upon titration with NaCl solution (0.2 M, titration step: 0.1 mL)

Spectroscopic studies of GNPs bifunctionalized with thiol derivatives of 13-membered azobenzocrown, showed that higher concentrations of Na^+^ ions had to be used as compared to K^+^ levels in the case of nanogold modified with 16-membered macrocycles. The first spectral changes were observed at NaCl concentrations of 29 mM and since interaction between **Au2.TA** and Na^+^ ions was observed already at concentrations of 36 mM, it was concluded that interactions of sodium ions with 13-membered azobenzocrown are responsible for spectral changes only within a narrow range of sodium concentrations (29–36 mM). The colloid was found not to interact with potassium ions within that concentration range. As spectral changes for all the studied compounds were similar, the result of a single experiment involving **Au2.TA.10** is presented (Fig. 9).

**Fig. 10:**
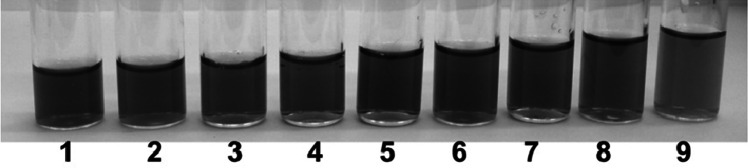
**Au1.TA.12** solutions (1.5 mL) at concentration of 0.36 mM containing increasing aliquots of 0.05 M KCl: *1* 0 mL; *2* 0.1 mL; *3* 0.2 mL; *4* 0.3 mL; *5* 0.4 mL; *6* 0.5 mL; *7* 0.6 mL; *8* 0.7 mL; *9* 0.8 mL. Photographs taken 5 min after addition of KCl solution

Simultaneously to spectrophotometric examinations, visual observations of non-modified GNPs, GNPs containing only lipoic acid and bifunctionalized GNPs in the presence of sodium, potassium, calcium and magnesium ions were carried out. Figure 10 presents a series of colloidal solutions containing **Au1.TA.12** at a concentration of 0.36 mM and increasing aliquots of 0.05 M KCl.

**Fig. 11:**
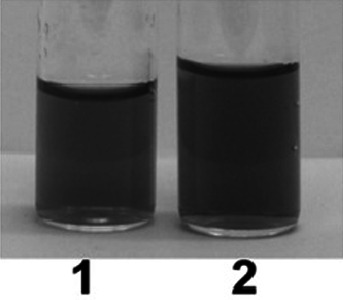
**Au1.TA.12** (1.5 mL) at concentration of 0.36 mM: 1) after addition of 0.3 mL of solution containing 8 × 10^−4^ M CaCl_2_, 8 × 10^−4^ M MgCl_2_, 1 × 10^−3^ M NaCl; 2) after addition of 0.3 mL of solution containing 8 × 10^−4^ M CaCl_2_, 8 × 10^−4^ M MgCl_2_ and 0.4 mL of 0.05 M KCl

Figure 11 compares the color of **Au1.TA.12** solution containing calcium, magnesium and sodium ions to the color of the same solution following the addition of potassium salt.

**Fig. 12:**
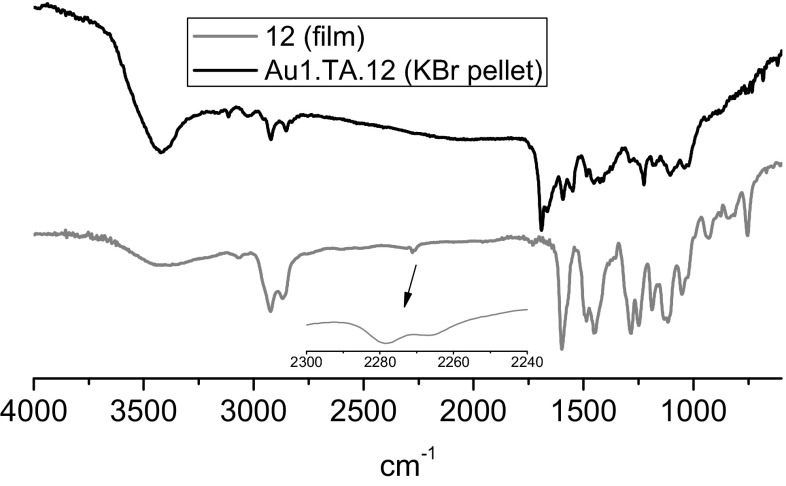
Comparison of FTIR spectra of azobenzocrown **12** and gold nanoparticles **Au1.TA.12** bifunctionalized with azobenzocrown **12** and lipoic acid

Infrared spectra were recorded for nanogold modified with lipoic acid and nanogold modified with both lipoic acid and azobenzocrown **12**. The spectra were compared with the spectra of azobenzocrown **12** and lipoic acid. Bands characteristic for both azobenzocrown and lipoic acid were found to be present in the IR spectrum of bifunctionalized nanogold (Fig. 12).

## Conclusions

Starting from 4′-hydroxyazobenzocrowns which can be synthesized in a reaction analogous to the Wallach rearrangement, as previously described by our group, a synthetic method was developed for the preparation of azobenzocrowns with terminal thiol group. Six new compounds with peripheral oxyalkane chains, with hydrocarbon chains of different length, terminated with thiol groups were obtained: three derivatives of a 16-membered and three derivatives of a 13-membered azobenzocrown. The peripheral chains were—O(CH_2_)_m_SH, where m = 2, 4, or 10. Novel, reactive azobenzocrown derivatives—six compounds with peripheral oxyalkane chains terminated with bromine were also obtained for the purpose of these syntheses.

The thiol derivatives of azobenzocrowns were used for preparation of nanosensors based on colloidal GNPs. Gold nanoparticles were functionalized with thiol azobenzocrowns and bifunctionalized with lipoic acid and thiol azobenzocrown derivatives being anchored to the gold surface. The systems were used in preliminary studies of the selectivity of interactions between alkali metal ions and modified colloidal GNPs. The assumption that nanogold modified with 16-membered azobenzocrown derivatives, analogs of which are known to form sandwich-type complexes with potassium ions and are successfully used as potassium ionophores in ISEs, would selectively react with potassium ions also when combined with colloidal gold was confirmed. As the result of the interaction, the color of the colloidal solution changed from burgundy to blue within the potassium ion concentration range of ca. 4–26 mM in the case of nanogold modified with thiol azobenzocrowns and within the potassium ion concentration range of ca. 8–20 mM for more stable, bifunctionalized nanogold. Lower concentrations of potassium can be detected when more dilute nanogold solutions are used. Most promising in terms of colorimetric detection of potassium ions in water is nanogold functionalized with a derivative featuring a C4 chain (compound **13**), affording a relatively fast and stable response. This compound is more stable and can be obtained with higher yield than derivative **12**. The results obtained for nanogold modified with 13-membered azobenzocrowns, i.e., sodium ionophores, were less encouraging as the change in the color in the presence of sodium ions was observed only at high concentrations of these ions (ca. 29 mM), too close to the threshold value above which the non-modified colloidal gold forms aggregates in the presence of sodium chloride (ca. 36 mM).
